# Biomedical articles share annotations with their citation neighbors

**DOI:** 10.1186/s12859-021-04044-4

**Published:** 2021-02-26

**Authors:** Raul Rodriguez-Esteban

**Affiliations:** Roche Innovation Center Basel, Roche Pharmaceutical Research and Early Development, 4070 Basel, Switzerland

**Keywords:** Biomedical database, Document annotation, Citation network

## Abstract

**Background:**

Numerous efforts have been poured into annotating the wealth of knowledge contained in biomedical articles. Thanks to such efforts, it is now possible to quantitatively explore relations between these annotations and the citation network at large scale.

**Results:**

With the aid of several large and small annotation databases, this study shows that articles share annotations with their citation neighborhood to the point that the neighborhood’s most common annotations are likely to be those appearing in the article.

**Conclusions:**

These findings posit that an article’s citation neighborhood defines to a large extent the article’s annotated content. Thus, citations should be considered as a foundation for future knowledge management and annotation of biomedical articles.

## Background

Biomedical annotations aim to categorize the knowledge contained in unstructured form in scientific articles and are at the core of many biomedical databases, such as MEDLINE’s Medical Subject Headings (MeSH) or UniProt’s literature-based data. While annotations have been widely used for many applications, the relation between annotations and the citation network has been underexplored.

Delbecque and Zweigenbaum [1] already pointed at the value of an article’s listed references by using them—among other article features—to automatically suggest MeSH term annotations for the article. Their analysis was based on full-text Journal of the American Medical Association (JAMA) articles published between the years 1998 and 2008. Perhaps due to lack of accessibility to normalized citation information, other studies in automatic annotation did not explore further the citation network for this task [2].

Recently, however, there has been a large increase in the availability of normalized citation information thanks to the Initiative for Open Citations (I4OC), which was established in 2017 [3]. The I4OC provides an open dataset of machine-readable references belonging to publications and which have been mapped to digital object identifiers (DOIs) to facilitate processing. The I4OC estimated that 59% of all Crossref citations had already become open by December 2020 (https://i4oc.org/#about; website checked on January 14, 2021). This surge in availability has enabled the large-scale use of citation information for the analysis of biomedical articles. Leveraging such data, for example, Rodriguez-Esteban [4] pointed out that terms with ambiguous meaning are typically not ambiguous within the network of articles directly connected by citations.

Through a quantitative assessment using several large and small annotation datasets, this study explores the relation of the citation network and biomedical annotations by measuring the degree to which articles tend to share annotations with their citation neighbors.

## Results

The information retrieval metrics precision and recall were used to measure the degree of annotation sharing between a MEDLINE article and its citation neighbors in several large annotation datasets (gene2pubmed, UniProt, MeSH and dbSNP). Recall, in this case, was the percentage of annotations in an article that appeared at least once in a neighbor. Precision was the percentage of unique annotations in the neighborhood that also appeared in the article. More intuitively, precision represented how frequently annotations in an article’s neighborhood were shared by the article. Recall went in the other direction: it represented how frequently annotations in an article were shared by its neighborhood. Together, precision and recall, define the overlap between an article’s annotations and those of its neighborhood.

**Fig. 1:**
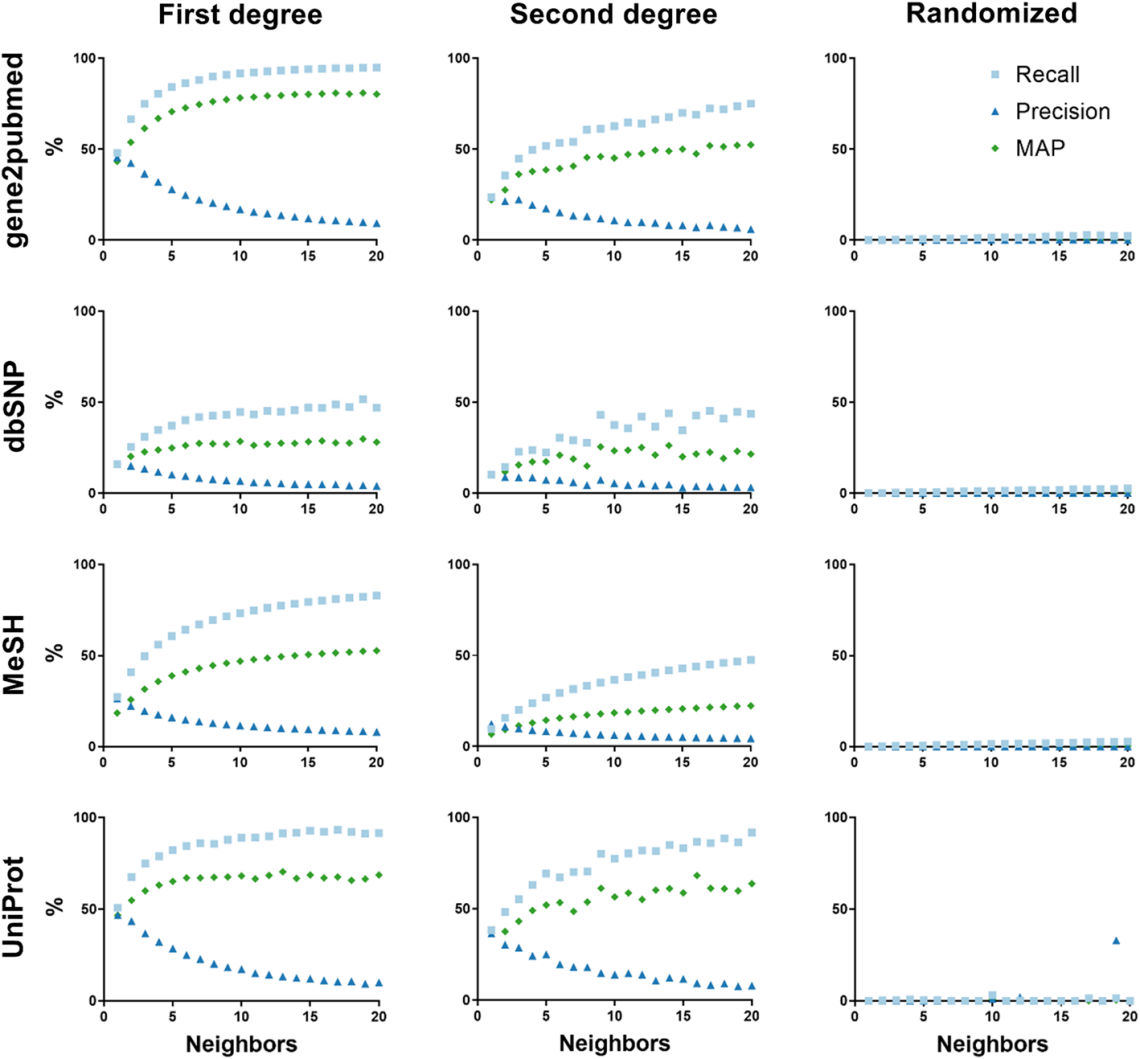
Recall, precision and mean average precision (MAP) for the gene2pubmed (first row), dbSNP (second row), MeSH (third row) and UniProt (fourth row) datasets for first-degree neighbors with annotations (first column), including second-degree neighbors with annotations (second column), and randomized network (third column)

Results showed that recall and precision depended on the number of existing annotated neighbors. For example, in gene2pubmed, average recall for articles with 3 annotated neighbors was 75% and increased with the number of annotated neighbors, reaching 95% with 20 annotated neighbors (arbitrary cut-off). The same could be observed with other datasets (Fig. 1). For example, with 3 annotated neighbors, average recall was 75% for UniProt, 49% for MeSH and 31% for dbSNP. For comparison, a randomized network of citations only led to average recall values < 1% for all datasets.

Precision ran in the opposite direction to recall. In the case of gene2pubmed, average precision was 46% for records with 1 annotated neighbor and decreased thereafter with the number of annotated neighbors. For Uniprot, MeSH and dbSNP average precision was 47%, 26% and 16%, respectively.

The metrics of recall and precision do not take advantage of annotation frequency information because they are based on unique annotations. Intuitively, however, annotations that appear more frequently in an article’s citation neighborhood should be more likely to be shared with the article. To test this hypothesis, neighborhood annotations can be ranked by annotation frequency and the quality of the ranking can be measured with a metric such as mean average precision (MAP). In the best-case scenario (MAP = 1), the most-frequent neighborhood annotations comprise the article’s actual annotations.

MAP values for gene2pubmed annotations increased with the number of annotated neighbors (Fig. 1) and reached 0.80 with 20 annotated neighbors, indicating that approximately 80% of the results at the top of the annotation ranking were shared between an article and its neighborhood. For MeSH, the MAP achieved was 0.53, while for dbSNP and UniProt it reached 0.28 and 0.69, respectively.

Obviously, MEDLINE articles for which no annotated neighbors were available could not be analyzed with this method, whether due to lack of annotation or of citation data. Only 14% of all MEDLINE records had at least one connection to a record annotated by gene2pubmed and 10% had two or more. Thus, the neighborhood of all annotated gene2pubmed articles covered only a selected fraction of MEDLINE. One way to reach articles not directly connected to annotated articles is to consider articles that are two steps away in the citation network. In fact, 44% of all MEDLINE had at least a connection to a first- or second-degree neighbor annotated in gene2pubmed and 40% had at least two connections. For UniProt, 31% of MEDLINE had at least one annotated neighbor and 26% had at least two. For dbSNP, it was 33% and 27%, respectively. Including second-degree neighbors in computing the MAP led to lower scores (Fig. 1), yet values still reached 0.53 in gene2pubmed, 0.27 in MeSH, 0.22 in dbSNP, and 0.64 in UniProt.

Performing this analysis over the entire MEDLINE does not reflect, however, how it applies in practice because the subset of articles that are the focus of annotation (e.g. articles that could be annotated with gene names in the case of gene2pubmed) is likely to be clustered within the citation network to some extent. A practical example can be demonstrated using the BioCreative II gene normalization (BC2GN) challenge dataset [5]. The BC2GN challenge was a competition of algorithms for automatic prediction of NCBI Gene ID annotations in MEDLINE abstracts that used a gold standard of 531 annotated abstracts. To analyze this dataset, annotation neighbors of the BC2GN articles are considered to be neighboring articles with potential gene2pubmed annotations.

**Fig. 2:**
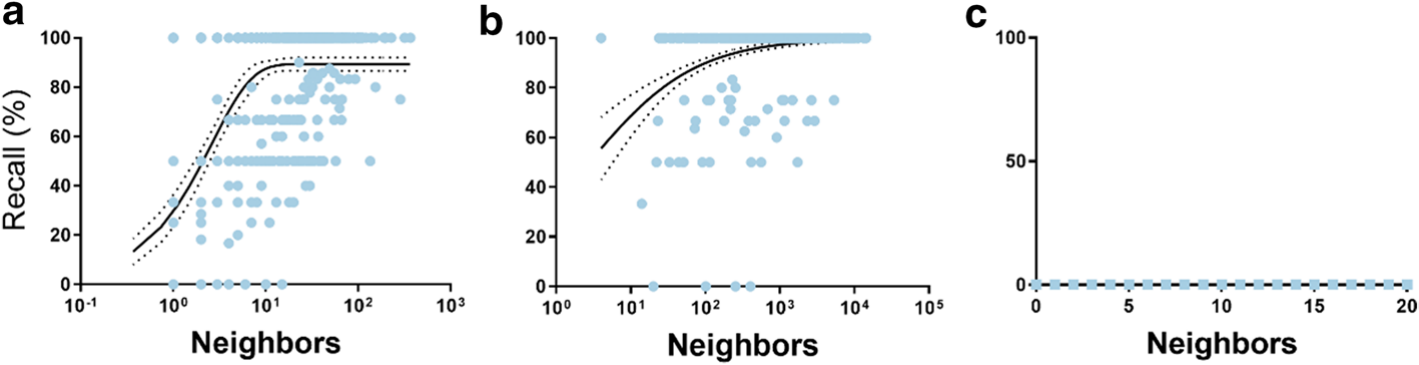
**a** Recall for BC2GN annotations based on the number of annotated neighbors. A sigmoidal function fit (*R*^2^ = 0.61) with 95% confidence intervals is shown alongside (dotted lines). The sigmoidal function was chosen because it is a monotonic function that is bounded within a range. **b** Including second-degree neighbors (sigmoidal function fit with *R*^2^ = 0.85). **c** For the randomized network

With our citation and annotation data, the BC2GN gold standard abstracts had a median number of 59 total connections and a median of 16 connections to neighbors with gene2pubmed annotations. Based on those neighboring annotations, 58% of the BC2GN records had 100% annotation recall. Moreover, 70% of all BC2GN annotations appeared in at least one neighbor’s gene2pubmed annotations, which means an overall corpus-wide 70% annotation recall (Fig. 2). Using a randomized citation network, instead, 0% of the records had 100% annotation recall and corpus-wide annotation recall was < 1%.

**Fig. 3:**
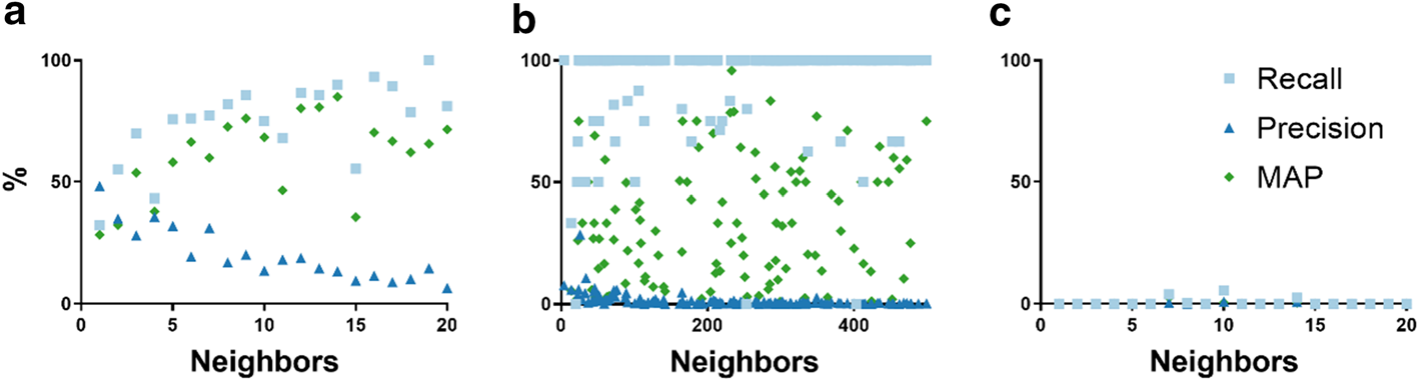
Recall, precision and MAP for the BC2GN dataset: **a** for first-degree neighbors with annotations, **b** including second-degree neighbors with annotations, and **c** for the randomized network. Values shown are noisy due to the low number of samples associated to each data point

Figure 3 shows the average recall, precision and MAP for BC2GN abstracts based on the number of annotated neighbors. As can be seen, recall and MAP generally increased with the number of annotated neighbors, while precision decreased. These numbers are consistent with the results already presented for the previous datasets. It is important to note that BC2GN and gene2pubmed annotations were made independently from each other and under different guidelines. Thus, results could have been higher if only annotations from a single source had been considered.

A hurdle for this analysis was the dearth of connections available for some BC2GN abstracts. For example, no connections were available for 85 abstracts (16%). An increase in the number of connections, however, would be expected to lead to higher recall (Fig. 2). In fact, we can extrapolate that approximately 76% recall could be achieved if all BC2GN articles had exactly 5 annotated neighbors. Extending the citation neighborhood of the BC2GN articles to include second-degree neighbors led to 82% of the abstracts reaching 100% recall and a corpus-wide 87% annotation recall. This was associated to a concomitant large increase in connections: the median number of annotated neighbors jumped to 1095 and the median total number of connections to 5532. However, 54 abstracts (10%) still had no connections.

**Fig. 4:**
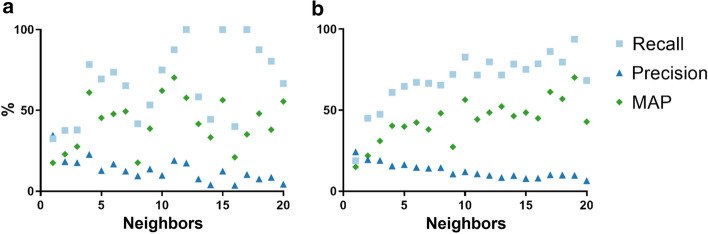
Recall, precision and MAP for MeSH term overlap analysis on the **a** NLM2007 and **b** L1000 datasets using first-degree neighbors with annotations. Values are noisy for small datasets, such as these, due to the low number of samples associated to each data point. Thus, values associated to NLM2007, with 200 articles, are noisier than for L1000, with 1000 articles

A further evaluation was done with the L1000 and NLM2007 datasets, which are reference datasets for MeSH term annotation [6, 7] (Fig. 4), showing similar overall results though nonetheless lower recall and MAP than for the BC2GN dataset. As can be seen in the figure, the smaller a dataset, the noisier the results.

**Fig. 5:**
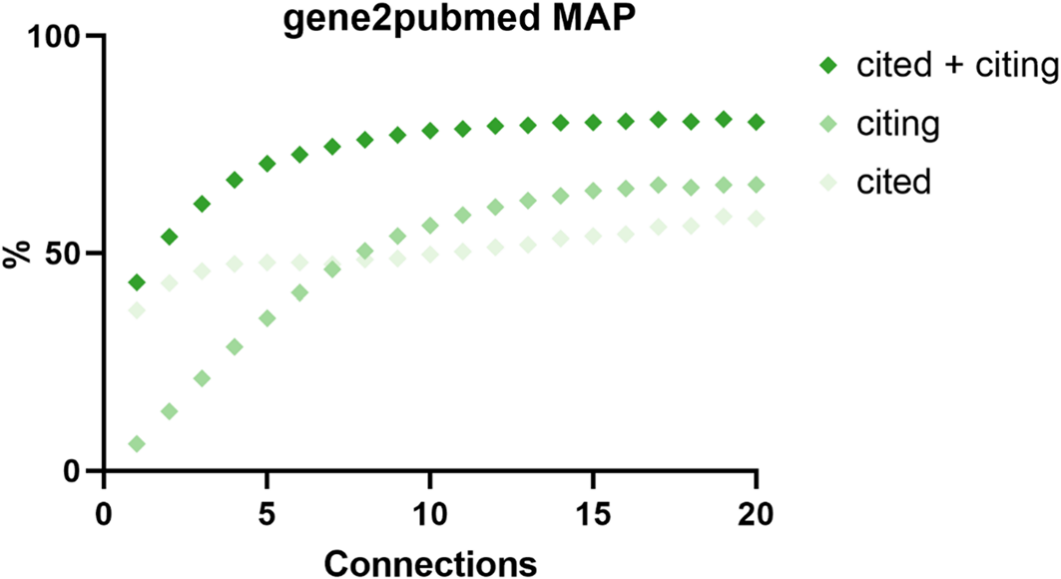
Mean average precision (MAP) for the gene2pubmed datasets for first-degree neighbors with annotations: **a** considering citing and cited references. **b** Considering citing references alone, and **c** cited references alone

The analysis presented thus far was done with an undirected citation network. If the direction of citations had been taken into account, results would have been lower, as can be seen in Fig. 5 for the gene2pubmed dataset. When either citing or cited references were taken into consideration, results were lower than when both were considered. Unsurprisingly, the value of citing references increased steadily with their availability, while the value of cited references (those appearing in the article itself) barely did. This demonstrates how the ranking improves as the amount of neighborhood annotation increases but it has limited dependency on the number of cited references.

## Discussion and conclusions

Overall, this study shows that an article tends to have similar annotations to those that are most frequent in its citation neighborhood, as expressed by the MAP metric. High recall values, on the one hand, show that citation neighbors tend to include most annotations present in an article. Low precision values, on the other hand, indicate that neighbors can include a diversity of annotations not shared by the article. As could be expected, annotation sharing is weaker when including articles two steps away in the citation network, leading to higher recall but lower precision and MAP. Additionally, while the recall achieved can be high, there is a remaining degree of novelty in individual articles with respect to their neighbors that allows for some differentiation.

The degree of annotation sharing found depended on the dataset considered, being highest—among the large datasets—for gene2pubmed and lowest for dbSNP. Smaller reference datasets showed similar properties. Thus, the degree by which the citation neighborhood defined the content of an article varied according to the type of annotation. The difference in level of annotation sharing across datasets could be due to differences in the cardinality of each annotation type. While the gene2pubmed dataset had 38,738 unique annotations, MeSH 28,993 and UniProt 18,931, the dbSNP dataset had 233,367. The diversity of dbSNP annotations could mean, therefore, a lower chance of overlap between neighboring annotations. Another factor influencing the degree of overlap could be annotation inconsistency, which would be more expected from annotations that are subject to a larger degree of annotator discretion (i.e. MeSH).

The small datasets (BC2GN, L1000, NLM2007) were used to show the practical implications of these results with respect to limited-size datasets, which could be of the size of a curation task with bounded timeline and resources [8]. Thus, while the coverage of current annotation databases is narrow with respect to the entire MEDLINE, it is much denser in neighborhoods of articles that are more likely to be relevant for annotation tasks.

A corollary to these findings is that annotations in an article’s citation neighborhood can be considered as features for automatic, semi-automatic or manual annotation methods—in the latter case with reasonable curation overhead [8]—for multiple types of annotations. It is interesting to note that web hyperlinks, which can be considered analogous to article citations, have already been used to annotate web document metadata under an approach called “metadata propagation” (e.g. [9–12]).

Delbecque and Zweigenbaum [1] already showed the utility of first-degree annotation neighborhoods to create features for machine learning by using a limited dataset (JAMA articles from 1998 to 2008) and one annotation type (MeSH). This study goes further in exploring annotation neighborhoods for first and second degree neighbors for different types of annotations (MeSH, dbSNP, UniProt, gene2pubmed) with a much larger set of publications and citations (MEDLINE and I4OC). Moreover, Delbecque and Zweigenbaum [1] did not explore how database annotation coverage and completeness could affect the utility of annotation neighborhoods.

While the goal of this study was not to develop a state-of-the-art document annotation algorithm based on citations, it can be of interest to compare the results presented here to those from other annotation methods, which are typically specialized in one annotation type alone. This is not a straightforward task because annotation predictions are not typically ranked and, therefore, the MAP is not used. One area in which MAP values have been used, however, is annotation of MeSH terms. The results reported here for MeSH annotations were above those from the Medical Text Indexer [from the National Library of Medicine (NLM)] for articles with 10 annotated neighbors or more (Fig. 4). Results were lower, however, than that of the more complex and specialized algorithms developed by [6, 13].

One could envision leveraging the findings of this study with an annotation database growing its article coverage simply by “following” selected article citations in order to cover a network of interconnected articles. Alternatively, selected highly-connected articles could be annotated as initial “seeds” of an annotation database, thereby facilitating the annotation of other articles connected to them. This would be an analogous strategy to that already adopted in protein structure resolution, in which key unique protein structures are manually selected for curation to maximize the reach of homology modeling [14]. Broadly speaking, using existing annotations to preliminary annotate articles could be considered a type of “annotation transfer,” in analogy to the prediction strategy for protein-coding DNA sequences by which newly-sequenced organisms are tentatively annotated based on knowledge from similar, already-characterized proteins from other organisms [15].

Limitations of this approach are based on the need for sufficient annotations and citation data for it to be effective. The latter particularly for articles that have never or seldom been cited because best results are achieved with the help of citing annotated articles. On the other hand, an advantage of the approach is that the context provided by neighborhood articles with similar annotations could be used to aid the work of manual annotators, in particular to increase consistency across articles within the same niche—or even to correct existing annotations based on inconsistency within neighborhoods.

While the datasets explored concerned single-concept annotations, other types of annotations (e.g. protein–protein interactions) could be explored if enough data were available. Existing citation data is abundant but still incomplete. Its availability, however, should increase with time, as more normalized citation data becomes available thanks to the Initiative for Open Citations (I4OC). One avenue of further exploration could be to use text similarity metrics instead of citations to determine which publications are most likely to share annotations with a publication of interest. Citation practice, after all, can be subject to a considerable degree of arbitrariness and field-specific biases [16, 17]. Textual similarity, particularly of full text content, could address such issues while still remain an approach that is simple to implement and interpret.

Finally, this study presents further quantitative evidence of topic specialization within the networks of articles connected by citations—e.g. scientific fields—and highlights the importance of connectivity to define the knowledge that is contained within documents, in this case by showing that neighborhoods of articles “collectively” define the content of the articles to which they are connected.

## Methods

This analysis considers scientific articles as the nodes of a network connected by citations, which are treated as undirected connections. Thus, each node is an article whose first-degree neighbors are other articles that cite it or that are cited by it. Second-degree neighbors are nodes that are two steps away in the network. The second-degree network comprises here both first-degree and second-degree neighbors.

The focus of the analysis was on scientific articles represented by MEDLINE records identified by their corresponding PubMed IDs (PMIDs). MEDLINE records came from the MEDLINE Baseline 2021. Citations came from the I4OC’s Open Citation Index repository [3], and in particular from the December 2020 update, which contained 759,516,585 citations between articles identified by a digital object identifier (DOI). DOI to PMID mappings were extracted from the European Bioinformatics Institute’s PMID-PMCID-DOI dataset [18] downloaded on January 13, 2021, which provided 23,407,831 mappings between PMIDs and DOIs. Using these mappings, 286,515,961 citations from the Open Citation Index were mapped from DOIs to PMIDs. Each citation was represented as a pair of PMIDs. The second-degree network comprised a total of 40,046,822,364 pairs of records. Citations were randomized by randomly shuffling the set of first PMIDs in all pairs of PMIDs.

Each node of the network was associated to annotations from four different databases (see dataset sizes in Table 1). Namely, two gene annotation databases (gene2pubmed, UniProt), one mutation database (dbSNP) and MeSH term annotations from the National Library of Medicine (NLM). Additionally, two gold standard annotation datasets for MeSH terms (NLM2007, L1000) and one for gene names (BioCreative II Gene Normalization (BC2GN)) were used. From the gene annotation datasets only human gene annotations were considered. gene2pubmed [19] is a dataset of gene name annotations maintained by the NCBI Gene database and was downloaded on January 13, 2021. UniProt data was based on UniProtKB [20], which was downloaded on January 13, 2021. The annotations used were those concerning “related publications,” which are publications associated to particular proteins. BC2GN [21] was downloaded on December 10, 2018 and comprised two files: the training set v1.4 with 640 annotations and the testing set v1.0 with 785 annotations. For Medical Subject Headings (MeSH) annotations, only those designated as “Major Topic” from the MEDLINE Baseline 2021 were used. The NLM2007 and L1000 gold standard datasets [6] of MeSH annotations contained, respectively, 200 and 1000 annotated records. Mutation annotations came from the dbSNP [22] b153 release downloaded on January 13, 2021 (unpacking of the newer b154 release led to errors). dbSNP is an archive of molecular variation information hosted by the National Center for Biotechnology Information (NCBI) with extensive literature references associated to particular variations.

**Table 1:** Dataset sizes

Dataset	Annotations	PMIDs
BC2GN	1425	531
dbSNP	506,325	87,911
gene2pubmed	1,572,276	677,822
L1000	3587	1000
MeSH	95,482,280	27,175,482
NLM2007	748	200
UniProt	213,618	78,471

The metrics used for evaluation were precision, recall and mean average precision (MAP). Precision is the number of true positives divided by all positives [TP/(FP + TP)] and recall the number of true positives divided by true positives and false negatives [TP/(TP + FN)]. In this case, recall was the percentage of annotations in a record that appeared at least once in a neighboring record. Precision was the percentage of unique annotations in the neighborhood that also appeared in the record. Mean Average Precision (MAP) is a metric widely used in information retrieval to evaluate rankings. For a ranked set of predictions, Average Precision (AP) is defined as:

$$AP = \mathop \sum \limits_{k = 1}^{N} P\left( k \right)\Delta R\left( k \right),$$

where *N* is the total number of predictions, *k* is the prediction’s rank, *P(k)* is the precision of all predictions up to rank *k* and *ΔR(k)* is the change in recall between predictions at rank *k* and at rank *k *− 1. MAP is the average AP over all sets of predictions.

This study can be reproduced with the code available here: https://github.com/raroes/annotation-sharing-in-biomedical-articles.

## Data Availability

The datasets analyzed during the current study are publicly available for BC2GN (https://biocreative.bioinformatics.udel.edu/tasks/biocreative-iii/gn/), dbSNP (ftp://ftp.ncbi.nih.gov/snp/), gene2pubmed (ftp://ftp.ncbi.nlm.nih.gov/gene/DATA/gene2pubmed.gz), L1000 (https://www.ncbi.nlm.nih.gov/CBBresearch/Lu/indexing/), MEDLINE (https://www.nlm.nih.gov/bsd/medline.html), MeSH (ftp://nlmpubs.nlm.nih.gov/), NLM2007 (https://www.ncbi.nlm.nih.gov/CBBresearch/Lu/indexing/), Open Citation Index (https://opencitations.net/), PMID-PMCID-DOI (ftp://ftp.ebi.ac.uk/pub/databases/pmc/DOI/), UniProt (ftp://ftp.uniprot.org/pub/databases/uniprot/current_release/knowledgebase/idmapping/idmapping_selected.tab.gz).

## Abbreviations

AP: Average precision
BC2GN: BioCreative II gene normalization
DOI: Digital object identifier
FN: False negatives
FP: False positives
I4OC: Initiative for Open Citations
MAP: Mean average precision
MeSH: Medical Subject Headings
PMID: PubMed ID
TN: True negatives
TP: True positives
